# Cardiovascular and Vector-Cardiographic Effects of Articaine Anesthesia with Epinephrine

**DOI:** 10.1155/2024/8610423

**Published:** 2024-06-14

**Authors:** Christoph Pökel, Antina Schulze, Martin Busse

**Affiliations:** ^1^ General Outpatient Clinic of Sports Medicine University Leipzig, Leipzig, Germany; ^2^ Department of Sports Dentistry Institute of Sports Medicine University Leipzig, Leipzig, Germany

## Abstract

The aim was to investigate the vector-cardiographic effects in patients submitted to dental extraction under local anesthesia. Twenty-one patients aged 36.6 ± 12.4 years with a clinical and radiographic indication of mandibular or maxillary tooth extraction were enrolled. The intervention was a local or mandibular nerve block anesthesia with 4% articaine hydrochloride containing epinephrine (1 : 100,000; 40 mg/ml + 10 *μ*g/ml). Blood pressure (BP), heart rate (HR), pulse wave transit time, and vector-cardiography data were recorded throughout 3 min before and 5 min after injection. *QRS*- and *T*-wave area under the curve (*QRS*_AUC_/*T*_AUC_) were calculated from the *X*/*Y*/*Z QRS*-vector or *T*-vector. *T*-wave amplitude (*T*_AM_), *T*_AUC_ values, and diastolic BP decreased, and HR significantly increased 4 min after injection. A transient moderate HR drop and a corresponding small increase in *T*_AM_ and *T*_AUC_ immediately after the injection procedure may be explained by a decreased sympathetic tone due to psychological relief. In dental anesthesia, the systemic epinephrine effects are represented by a decrease in *T*_AUC_. These effects are most pronounced in the *X*- and *Y*-leads. The 3D determination of vector planes or amplitudes is a simple method to register the sympathetic tone in local anesthesia independently of possible effects on *T*-wave characteristics in single leads. In conclusion, *T*-wave determination may help to detect even small increases in systemic adrenaline concentration in case of accidental intravascular injection. At the same time, full rhythm and spatial ischemia control is provided.

## 1. Introduction

Epinephrine is the most used vasoconstrictor in association with local anesthesia in dentistry. The epinephrine content offers advantages, such as rapid onset, depth and duration of anesthesia, less local blood loss, and decreased systemic absorption of the anesthetic [1]. Approximately 20% of all intraoral injections are likely to result in at least briefly elevated systemic adrenaline levels [2]. Physiological responses associated with local anesthetics containing epinephrine showed changes in HR and BP [3, 4], dysrhythmias [5, 6], changes in ST-segment and *T*-wave [7, 8], and release of endogenous catecholamines [9]. Systemic side effects of local anesthesia may be tachycardia, ischemia, or increased blood pressure (BP) and/or cranial nerve involvement and temporary palsy [4, 10, 11, 12, 13]. An increased occurrence of extrasystoles was the most frequently described side effect [14, 15]. Also, ST-segment changes [14] and cardiac arrhythmias [14, 15, 16] were observed. Though cardiovascular complications in dental anesthesia are rare, the incidence of 4.5% or 5.7% of risk patients, respectively, [17] should not be neglected. Whether isolated cardiac arrhythmias are dangerous for a patient depends primarily on the patient's age and personal state of health [15]. Increased-risk patients, identified by their medical history, can be found in the daily dental practice and may require special attention, such as monitoring of heart rhythm and *T*-wave changes. In addition, there is a considerable number of undiagnosed cases in the field of cardiovascular diseases [18].

The majority of cardiac problems mentioned above can be detected in an ECG. While a 1-lead ECG is sufficient for arrhythmia detection, a complex 12-lead ECG would be required for all de- and repolarization disturbances. The vector ECG is an easy-to-handle alternative that spatially records the heart in three leads. 3D vector-cardiography (VCG), in addition, offers the distinct advantage of quantitatively determine the spatial *T*-wave area or amplitude. *T*-wave amplitude (*T*_AM_) is known to be influenced by the parasympathetic nervous system [19] and the sympathetic nervous system [20] and is an important measure of ventricular tachyarrhythmia [21].

The present study aims to show for the first time, which effects of local anesthesia can be seen on excitation formation (*QRS*-complex) and repolarization (*T*-wave) in the special vector ECG of patients without cardio-circulatory disease under the conditions of dental anesthesia prior to a tooth extraction.

## 2. Materials and Methods

### 2.1. Study Participants

Twenty-one participants (2 females; 19 males) aged 21–74 years (mean: 36.6 ± 12.4 years) without structural heart disease, selected at the Department of Sports Dentistry at the Institute of Sports Medicine of the University of Leipzig were enrolled in this study. The study was approved by the Ethical Review Board of the Medical Faculty of the University of Leipzig (No. 194/2019) and was in accordance with the Helsinki Declaration. A written informed consent was obtained from all patients. To be included, patients had to present at least one molar or premolar (regardless of superior or inferior) with one of the clinical and radiographic indications of extraction: coronal destruction, presence of furcation perforation, extensive apical periodontitis, and root residues. All patients were examined clinically and radiographically. Patients with acute pain, analgetic medication, platelet disorders, cardiovascular disease, diabetes mellitus, tricyclic antidepressant treatment, allergic or hypersensitivity reactions to anesthetics, asthma, or liver disease were excluded.

### 2.2. Measurements

For the VCG, careful skin preparation was performed to reduce the impedance between the skin and electrodes. For vector ECG analysis, a vector cardiographic recording (Cardiax PC-EKG, MESA Medizintechnik, Germany) was made continuously with the orthogonal leads (*X*/*Y*/*Z*), according to Frank [22]. The magnitude of a vector can be defined as the height (amplitude) or the distance between the initial point and the endpoint of a vector or the area of the *T*-wave. Using a special software of the Cardiax PC ECG, the *QRS*- and *T*-wave area under the curve (*QRS*_AUC_, *T*_AUC_) and amplitude (*T*_AM_) were automatically determined. For the calculation of *QRS*_AUC_ and *T*_AUC_ the integrals under the *QRS*- and *T*-waves of leads *X*, *Y*, and *Z* were averaged over 10 s and the *QRS*/*T*-vector magnitude was calculated as *QRS*_AUC_/*T*_AUC_ = √ (*TX*^2^ + *TY*^2^ + *TZ*^2^), where *QRSX*/*TX*, *QRSY*/*TY*, and *QRSZ*/*TZ* are the averaged *QRS*-/*T*-wave area of each *X*-, *Y*-, *Z*-lead. The ECG was averaged every ten seconds by forming an overall vector of the *X*-, *Y*-, and *Z*-leads.

Hemodynamic recordings were systolic and diastolic blood pressure (SBP, DBP) and heart rate (HR). VCG was recorded continuously as soon as the patient was seated in the treatment chair. From the ECG-curves, the pulse wave transit time (PWTT) was determined [23]. Upper arm BP measurements were taken every minute using an automatic BP measuring equipment (Boso-Medicus, Bosch & Sohn, Germany). After the baseline VCG was recorded for a minimum of 3 min, all patients received local anesthesia as described in the following paragraph, which was injected as a local infiltration or inferior alveolar nerve block, dependent on the tooth location. The measured values of the 3-min resting period represented the baseline values (BVs). After the injection of the anesthetic solution, the tooth extraction started after a period of at least 5 min. This 5-min period was divided into minute-intervals from the first to the fifth minute after injection (AI). The sum of the time periods BV and 1AI to 5AI resulted in a total examination time of 8 min. For data analysis, the measured parameters at the different time points from 1AI to 5AI after anesthesia were compared with each other and with the BVs.

### 2.3. Dental Anesthesia

One single dentist performed all extractions. The dentist prepared the carpule syringe with a short or long needle for the tooth extraction in the upper or lower jaw (5 inferior alveolar nerve blocks and 16 local infiltrations). All patients with local infiltration received the dose of one anesthetic glass cartridge (1.7 ml) of articaine hydrochloride with epinephrine (3M/ESPE, Germany; 1 : 100,000; 40 mg/ml + 10 *μ*g/ml). A buccal infiltration local anesthetic technique was performed in the upper jaw between the roots of the (pre-) molars. The inferior alveolar nerve block technique was used for the anesthesia in the lower jaw with one cartridge articaine. When a molar surgery in the lower jaw was necessary, a quarter of the anesthetic cartridge was additionally injected to achieve a buccal nerve anesthesia (total articaine dose for alveolar nerve block and buccal nerve anesthesia: 2.2 ml). The injection of the anesthetic fluid was performed carefully with aspiration. The mean dose was 1.82 ± 0.22 ml. All patients were previously weighted (mean: 78.4 ± 15.8 kg).

### 2.4. Statistical Analysis

All results are expressed as mean ± standard deviation (SD). The significance of differences was calculated using an analysis of variance for repeated measures (one-way ANOVA test). Effect size (ETA-squared) was also calculated. GraphPad Prism version 9.4.1 was used for the statistical analysis and graphical representation. A *p*-value < 0.05 was considered significant.

## 3. Results

The main recordings are displayed in Table 1.

In relation to the BV (72.3 ± 11.9 bpm), HR decreased insignificantly in the 1 min after the anesthetic injection (1AI = 71.1 ± 13.5 bpm), followed by a significant increase until the 5th minute (5AI = 75.3 ± 10.4 bpm, *p* < 0.04).

No significant differences were observed for the SBP values over the study period. In contrast, the DBP decreased significantly from the 2nd minute (2AI) and remained significantly lower in the observation period when compared to the BV value. Mean BP changes were not significant.

Like SBP, no significant differences were found in PWTT or *QRS*_AUC_. In contrast, *T*_AM_ and *T*_AUC_ values changed significantly. *T*_AM_ was not significantly increased at 1AI but then decreased markedly in the following minutes. Each of the anesthetic techniques (infiltration (1.7 ml; 17 *μ*g epinephrine) and blockade of the inferior alveolar nerve (2.2 ml; 22 *μ*g epinephrine)) produced significant *T*-wave decreases without a significant difference between the techniques. However, there was a descriptive tendency towards a more pronounced flattening of the *T*-wave 5 min after the nerve block anesthesia (*ΔT*_AM_: −6.2% vs. −9.02% and *T*_AUC_: −8.1% vs. −18.5%).

**Figure 1:**
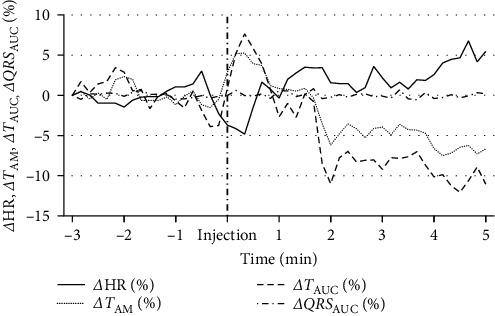
Changes in HR, *T*_AM_, *T*_AUC_, and *QRS*_AUC_ values within the observation period of 3 min before and 5 min after the injection, expressed as percentage and displayed as *Δ*HR, *ΔT*_AM_, *ΔT*_AUC_, and *ΔQRS* in 10 s intervals.

Significant changes in *T*_AM_ values were measured at 4AI and 5AI (*p* < 0.0001). The *T*_AUC_ values changed similarly during the investigation period (*p* < 0.0001). The differences in HR, *T*_AM_, and *T*_AUC_ values within the observation period of 3 min before and 5 min after the injection are expressed as percentages and displayed as *Δ*HR, *Δ*T_AM_, and *Δ*T_AUC_ in 10 s intervals in Figure 1.

**Figure 2:**
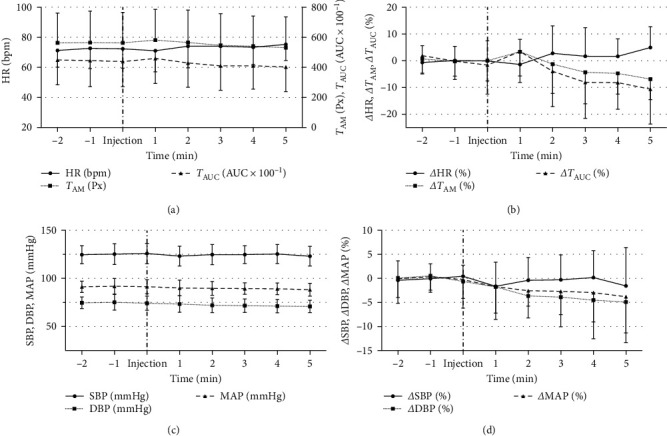
Cardiovascular and cardiographic parameters: absolute values (a, c) and changes (*Δ*) in comparison to baseline values (b, d) with an indication of the standard deviation. HR, heart rate; *T*_AM_, *T*-wave amplitude; *T*_AUC_, *T*-wave area under the curve; SBP, systolic blood pressure; DBP, diastolic blood pressure; MAP, mean arterial blood pressure.

The absolute values (*A*, *C*) and relative changes (*Δ*) in comparison to BVs of HR, *T*_AM_, *T*_AUC_, mean arterial pressure (MAP), SBP, and DBP values within the measured 8-min period are shown in Figure 2.

**Figure 3:**
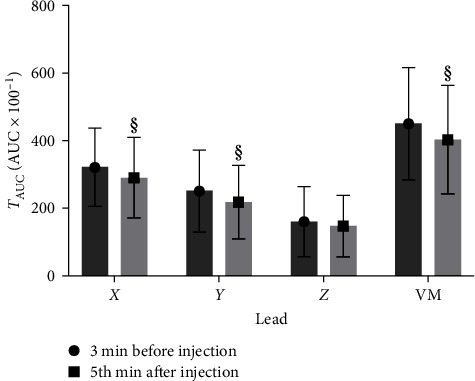
*T*-wave area under the curve (*T*_AUC_) in the *X*-, *Y*- and *Z*-leads and combined *X*/*Y*/*Z T*-vector magnitude 3 min before and 5 min after anesthetic injection. §: significant.

The *T*_AUC_ values measured 3 min before and 5 min after injection were separated for each lead (*X*, *Y*, *Z*) and presented in Figure 3. Significant differences were found in the *X*-leads (*p* < 0.0001), *Y*-leads (*p* < 0.0002), and combined *X*/*Y*/*Z T*-vector magnitude (VM; *p* < 0.0001). The *Z*-leads changed insignificantly.

## 4. Discussion

In the current study, three factors had a potential effect on the electro-cardiographic *T*-wave magnitude:

(1) Local anesthetics, (2) injection stress, and (3) epinephrine.

1. Articaine: Local anesthetics affect transmembrane ionic currents and, therefore, in principle, could also have an intrinsic effect on *T*-wave characteristics that are independent of sympathetic tone. A quicker onset, shorter elimination time, and low degree of toxicity are the advantages. Articaine is quickly metabolized into inactive articainic acid in plasma and tissues [24]. The local saturation of the serum esterases might contribute to the very low systemic toxicity [25]. Maximum blood concentration of articaine occurs about 10–15 min after submucosal application, irrespective of epinephrine [25]. A mandibular nerve block with 2 ml 4% articaine 1 : 200,000 in 20 patients showed an average maximum concentration of 2.1 ± 1.3 mg/l after 12.5 ± 2.5 min in peripheral blood samples [26]. In the current study, the analyzed time-period was limited to 5 min after injection and, therefore, to half or one-third of the time to reach maximum blood concentration. In a canine model, articaine-induced concentration-dependent changes in action potential, including shortening of the action potentials, reduction of their amplitude and maximum velocity of depolarization, suppression of early repolarization, and depression of plateau [27], occurred. These effects were seen only at concentrations higher than the therapeutic range, which can be achieved only by accidental venous injection [27]. In the present study, due to the local injection in combination with epinephrine with respective small vessel contraction, only very low plasma concentrations would be expected. Also, strict attention was paid to proper aspiration before and during injection. Thus, an intravascular injection could most likely be excluded. Overall, an effect of articaine on the *T*-wave can be excluded in the present study.
2. Injection stress: The mental stress due to the upcoming injection means an activation of the sympathetic nervous system [28]. The *T*-wave amplitude (*T*_AM_) as a psychophysiological index for the sympathetic drive has been sustainably reviewed. A *T*-wave decrease as a cardiac sympathetic index has been reported due to mental, psychophysical [29, 30, 31], and psychoneuro-cardiological [32] causes, anxiety [33], or cardiac nerve stimulation [34], also due to exercise-induced sympathetic increase [35] and catecholamine injection [33]. Mitchell and Shapiro [33] showed that symptoms of anxiety due to venous or arterial puncture in single patients produced ST-depression and *T*-wave inversion similar to the injection of adrenaline. In the current study, no anxiety scale was used. However, it can be assumed that the anxiety caused by the expectation of the injection disappeared immediately after the end of the injection. The slight decrease in HR and BP values from BV to 1AI can be interpreted in this direction. This corresponds with a slight increase in AUC, in line with the literature cited above [29, 30, 31]. Therefore, the early moderate HR drop and corresponding small increase in *T*_AM_ and *T*_AUC_ may be explained by a reduced sympathetic drive after injection.
3. Epinephrine: In general, the requirement is to use the minimum possible concentration of epinephrine, depending not only on the respective indication but also on the individual circumstances and tolerance [36], which should be evaluated prior to treatment. Approximately 20% of all intraoral injections are likely to result in at least briefly elevated adrenaline levels [2], which may be clinically relevant, because even low concentrations affect cardiovascular functions [37]. Thus, it has been reported that the occurrence of systemic complications in dental anesthesia is mainly based on the vasoconstrictor addition [38]. These potential complications include not only general malaise as well as cardiovascular problems but also ischemic events [4], tremors, hyper- or hypoglycemia, and potentiation of other drug-induced side effects [39, 40]. Basically, there are two main possible mechanisms of action of epinephrine: (a) Directly via sympathetic receptors and (b) Indirectly via influencing potassium homeostasis.

Ad (a) Epinephrine is an agonist on alpha, beta-1, and beta-2 receptors. The alpha-adrenergic agonist effect is vasoconstriction, which supports the analgetic effects in the tissues with limited effects upon BP [41]. Peak influences of epinephrine are generally observed within 5–10 min following injection [1]. Dionne et al. [42] showed that a submucosal infiltration of three cartridges of 2% lidocaine with epinephrine 1 : 100,000 increased cardiac output, HR, stroke volume, and SBP, while the MAP changed insignificantly. Hersh et al. [1] found similar results with 4% articaine containing epinephrine.

In the current study, HR decreased slightly by 1.2% in 1AI and then increased significantly by 3.0% in 5AI. In a comparable study, plasma epinephrine levels showed no increase within the first minute following anesthetic infiltration, but significantly higher plasma catecholamine levels were detected after the second minute [43].

The SBP did not change significantly. This is consistent with a previous study in 60 adults [15] and in 14 healthy adults [1]. Previous studies with respect to anesthetics containing epinephrine have confirmed that even though BP and HR may have changed significantly, the hemodynamic response, as defined by the MAP, stayed unchanged [44, 45, 46, 47].

In the current study, the sum of all Frank leads (*X*/*Y*/*Z*) was used as an independent measure of whole heart electric potential changes. According to Antzelevitch [48], the morphology of the *T*-wave is determined by three main factors: The magnitude of transmural dispersion of repolarization, the action potential duration of the three principal cell types in the epi- or endocardium and a “middle layer,” the M-cells, and the degree of electronic coupling between these layers. The de- and repolarization properties of special M-cells may play a major role in this context [48]. However, the M-cell theory is not generally accepted [49]. Though the physiology of the *T*-wave of the ECG is unclear [49], a transmural dispersion of repolarization due to varying catecholamine concentrations is the most reasonable explanation.

Ad (b) In the present study, 17 and 22 *μ*g epinephrine were injected. According to Knoll-Köhler et al. [43], the injection of 20 *μ*g epinephrine produced a doubling of plasma epinephrine levels and an increase of HR of about 4 bpm after 5 min, which is all near to our measured values. Theoretically, an indirect mechanism of *ß*-receptor stimulation may be a decrease in plasma potassium values [50]. Low plasma potassium may cause *T*-wave flattening or inversion [51]. However, only a high dosage (>40 ng/kg/min) of epinephrine decreases plasma potassium with a corresponding HR increase of more than about 15 bpm [52]. The highest HR increase after local anesthesia in the current study was about 5 bpm, indicating only a small plasma increase of epinephrine, and no relevant plasma potassium increase was expected [43]. In summary, small extrinsic increases in plasma epinephrine concentration were related to a clear and significant decrease in *T*_AUC_ and *T*_AM_. Even the slightly higher epinephrine dose of the alveolar nerve blockade showed a more pronounced decrease in *T*-wave characteristics than the infiltration anesthesia. However, this difference was not significant; the number of patients with nerve block anesthesia was low, and the results must be interpreted with caution.

In contrast, no effects on the *QRS* complex have been seen in the current study. Middlehurst et al. [53] reported *QRS* changes after local lidocaine application in conjunction with vasoconstrictors; however, the results on *QRS* have only been used to analyze dysrhythmia. Studies on electrophysiological effects of articaine in rabbits compared with bupivacaine and lidocaine basically showed effects on myocardial action potentials [54]. Overall, however, the articaine effects were small, even at higher doses in these animal experiments. This is confirmed by studies on the risk of local dental anesthesia [55], which reported no *QRS*-changes. The present study shows that even minimal changes in the *QRS* area (*QRS*_AUC_) are not detectable under the given dosage.

For clinical purposes, *T*-wave amplitude recording (as an alternative for the *T*_AUC_) has been recommended to monitor increases in plasma epinephrine, e.g., to detect intravascular injection of epinephrine [56, 57, 58]. Repolarization differences along the anatomic axes contribute to the *T*-wave [49], i.e., the *T*-wave area magnitude. The present study shows that the most pronounced response is achieved from the combined *X*/*Y*/*Z T*-vector magnitude and that this method is best to calculate *T*-vector changes independently of physiological effects on singular ECG leads.

For all critical cardiovascular cases in dentistry, ECG monitoring for rhythm control or repolarization abnormalities can be recommended. Neither the fundamentally required, careful cardio-circulatory, metabolic, or pulmonological history nor medical reports can predict the individual effect of local anesthesia. This is particularly true for patients whose cardiovascular disease is yet unknown. The automatic determination of the spatial ECG and especially the *T*-vector magnitude as vector area or vector amplitude is a simple method that is independent of possible physiological effects on the *T*-wave in single leads but includes all single lead information. The current study shows, first in patients without a cardio-vascular history, that even very low increases in a sympathetic tone, such as mental stress or a moderate increase in plasma catecholamines, can be detected by special vector ECG registration. This marks the high sensitivity especially of this method, in addition to the known importance for rhythm or ischemia detection. It is an important clinical conclusion that systemic overdosage and accidental intravascular injection could rapidly be detected in the same way. The fact that cardiac side effects of oral injection of local anesthetics are rare, even in the presence of existing cardiovascular disease, should not fundamentally question the ECG-monitoring in these patients. Systemic complications during dental treatment have been seen in 4.5% of all cases and 5.7% in patients with a disease history [17]. Cardiac safety in dentistry is, therefore, also a relevant cardiology topic. To illustrate the clinical-cardiac significance, the measures, and safety basics during stress ergometry, one of the particularly important cardiology tests, should be recognized. The 2002 American Heart Association exercise testing guidelines cite an adverse event rate of up to 0.04% or 1 per 2,500 tests for coronary artery disease [59]. Fatal adverse events by a rate for all-cause death in patients with severe left ventricular dysfunction are <0.1 per 1,000 ergometer tests (0.01%), and the rate of nonfatal cardiovascular events <1.0 per 1,000 tests (0.1%) [60]. Despite a rate of side effects that is lower than that cited for local oral anesthesia [15], a 12-lead ECG is mandatory for any ergometry in addition to other controls [61].

Hill et al. [62] used a 12-lead ECG to study the incidence of ECG abnormalities in healthy adults undergoing a surgical extraction of third molar teeth pre- and postoperatively. Even though no ECG recordings were taken during surgery, 44 from 198 patients showed ECG abnormalities. In patients with cardiovascular diseases, such events can lead to more severe problems. Ryder [6] reported that cardiac arrhythmia was not dose-related in 9.2% of the cases. Blinder et al. [63] found new ECG abnormalities in 37.5% of patients with known heart disease. Asymptomatic ischemic events are also reported [64]. Although local anesthesia is basically considered safe also in cardiac patients [16, 55, 65, 66], safety should still be improved by adequate monitoring [53]. The question of which patient groups are eligible for advanced cardiac vector-ECG recording should be based more on the fundamental hazards of special cardiac diseases and less on the general frequency of adverse events in the oral application of local anesthetics. Patients with unstable angina, recent myocardial infarction, decompensated heart failure, significant dysrhythmia, or severe valvular disease are at particular risk [67], and their safety can be improved by improved ECG monitoring.

Due to the specifics of its dental application, 3D VCG has more advantages than any conventional ECG recording. The technical effort is lower, the results can be presented in a trend analysis that requires almost no ECG knowledge, and in addition to the usual ECG-warnings, an accidental intravascular injection can be detected at an early stage.

In conclusion, for the first time, the effects of a dental anesthetic injection in cardiac-healthy patients on the vector ECG and especially on the *T*-wave have been studied. According to these initial results, area or amplitude measurement of the *T*-wave may be suitable for detecting even small increases in systemic adrenaline concentration. In addition to the detection of arrhythmia and ischemia, this may allow early detection of accidental intravascular injections. The study demonstrates the potential of VCG to provide a complex safety strategy with simple tools. A future aim is to record the vector-electrocardiographic abnormalities from a preventive point of view in a multicenter approach. This would complement the systemic management of cardiovascular risk patients in dental anesthesia [68]. Ultimately, a conscientious and complete medical history is necessary to identify possible risk factors as early as possible. Potential interactions with the patient's permanent medication can only be calculated if an updated list is available. Furthermore, the limited quantity of the used anesthetic solution must be strictly observed in every case to avoid accidental overdosage treatment.

## Figures and Tables

**Table 1:** Cardiovascular and vector-cardiographic parameters determined before and their change (*Δ*) in the 5-min period after anesthetic injection.

Parameter	Measured time points							
	BV	1AI	2AI	3AI	4AI	5AI	*p*-Value	*η* ^2^
HR								
bpm	72.3 ± 11.9	71.1 ± 13.5	73.9 ± 13.9	74.0 ± 13.3	73.4 ± 11.2	75.3 ± 10.4^§^	0.027	0.135
* Δ*HR (%)	0	−1.4 ± 7.1	2.8 ± 8.8	1.7 ± 9.1	1.6 ± 5.2	4.9 ± 6.0^§^	0.017	0.156
SBP								
mmHg	125.3 ± 9.5	123.1 ± 10.2	124.8 ± 10.5	124.8 ± 9.3	125.3 ± 9.9	123.1 ± 10.2	n.s.	0.044
* Δ*SBP (%)	0	−1.6 ± 4.4	−0.4 ± 4.0	−0.3 ± 4.9	0.2 ± 5.0	−1.6 ± 6.3	n.s.	0.043
DBP								
mmHg	74.7 ± 6.6	73.4 ± 8.6	72.0 ± 7.7^§^	71.7 ± 7.0^§^	71.2 ± 6.8^§^	70.9 ± 6.5^§^	0.016	0.175
*Δ*DBP (%)	0	−1.8 ± 6.1	−3.6 ± 4.5^§^	−3.9 ± 5.1^§^	−4.5 ± 6.4^§^	−4.9 ± 6.6^§^	0.024	0.162
MAP								
mmHg	91.5 ± 6.7	90.0 ± 8.1	89.6 ± 7.2	89.4 ± 6.0	89.2 ± 6.1	88.3 ± 6.6	n.s.	0.122
*Δ*MAP (%)	0	−1.7 ± 4.6	−2.5 ± 2.8	−2.7 ± 3.7	−3.0 ± 4.8	−3.8 ± 5.7	n.s.	0.132
PWTT								
ms	281.1 ± 28.5	279.6 ± 28.9	284.5 ± 26.2	280.6 ± 23.7	282.4 ± 25.7	274.2 ± 25.4	n.s.	0.123
*Δ*PWTT (%)	0	−0.5 ± 3.3	2.4 ± 5.2	0.2 ± 3.7	0.7 ± 3.2	−2.4 ± 6.8	n.s.	0.152
*T*_AM_								
Px	564.6 ± 205.6	581.9 ± 206.7	562.5 ± 216.0	547.8 ± 214.7	541.1 ± 203.2^§^	531.3 ± 208.6^§^	0.0001	0.309
*ΔT*_AM_ (%)	0	3.4 ± 8.0	−1.2 ± 8.2	−4.4 ± 10.1	−4.7 ± 6.2^§^	−6.9 ± 6.8^§^	0.0001	0.306
*T*_AUC_								
AUC ( ^*∗*^1,000)	44.5 ± 16.9	46.0 ± 16.6	42.7 ± 16.1	41.2 ± 16.7	41.1 ± 15.6^§^	40.3 ± 16.0^§^	0.0001	0.405
*Δ*AUC (%)	0	3.5 ± 8.6	−4.0 ± 9.1	−8.2 ± 11.9^§^	−8.2 ± 7.8^§^	10.6 ± 10.8^§^	0.0001	0.402
*QRS*_AUC_								
AUC ( ^*∗*^1,000)	59.5 ± 11.0	59.6 ± 11.0	59.6 ± 11.0	59.6 ± 11.2	59.5 ± 10.9	59.5 ± 11.0	n.s.	0.009
*Δ*AUC (%)	0	0.2 ± 0.3	0.1 ± 1.4	0.0 ± 0.8	−0.1 ± 1.3	0.0 ± 1.1	n.s.	0.009

BV, baseline mean values of the 3-min period before injection; 1AI–5AI, minute 1–5 after injection; HR, heart rate; bpm, beats per minute; SBP, systolic blood pressure; DBP, diastolic blood pressure; MAP, mean arterial blood pressure; PWTT, pulse wave transit time; *T*_AM_, *T*-wave amplitude; *T*_AUC_, integral of the area under the *T*-wave curve; *QRS*_AUC_, integral of the area under the *QRS* curve; *η*^2^, effect size (ETA); *p*-Value, significance value of the one-way ANOVA with repeated measures; §: significant difference compared to baseline values.

## Data Availability

We support the sharing of data to verify and replicate the results and perform secondary analysis if required. If the article is accepted for publication, the raw data can be provided in the form of a spreadsheet (.xlsx, .csv) upon request.

## Additional Points

*Limitations of the Study*. The current study was performed in a small collective of cardiac-healthy patients, especially with a low proportion of women. Thus, statements on patients with specific cardiac diseases are not yet possible. Upcoming studies in healthy subjects and patients with cardiac disease should further show to what extent the 3D VCG can also be used over a longer measurement period in conjunction with measured plasma catecholamine concentrations as a sensitive early indicator of excitation-formation and -recovery disorders.
